# Historical tree phenology data across contrasting sites in the Congo Basin

**DOI:** 10.1038/s41597-025-06542-z

**Published:** 2026-01-07

**Authors:** Koen Hufkens, Elizabeth Kearsley, Piet Stoffelen, Steven B. Janssens, Camille Couralet, Margaret Kosmala, Emmanuel Kasongo Yakusu, Donatien Musepena, Dieu-Merci Assumani, Nestor K. Luambua, Benjamin Toirambe, Jean-Remy Makana, Corneille Ewango, Tom De Mil, Marijn Bauters, Wannes Hubau, Elasi Ramanzani Kitima, José Mbifo Ndiapo, Adelard Lonema Chuda, Andrew D. Richardson, Lisa Wingate, Bhély Angoboy Ilondea, Hans Beeckman, Jan Van den Bulcke, Pascal Boeckx, Hans Verbeeck

**Affiliations:** 1 BlueGreen Labs, Melsele, Belgium; 2 Meise Botanic Garden, 1860 Meise, Belgium; 3 Leuven Plant Institute, Department of Biology, KU Leuven, Leuven, Belgium; 4 University of Montpellier, Montpellier, France; 5 Department of Organismic and Evolutionary Biology, Harvard University, Cambridge, MA USA; 6 CIBO Technologies, Cambridge, MA USA; 7 UGent-Woodlab (Laboratory of Wood Technology), Department of Environment, Ghent University, Ghent, Belgium; 8 Service of Wood Biology, Royal Museum for Central Africa, Tervuren, Belgium; 9 Université de Kisangani, Kisangani, Democratic Republic of the Congo; 10 Institut National pour l’Etude et la Recherche Agronomiques-INERA, BP 28 Yangambi, Democratic Republic of the Congo; 11 Ministère de l’Environnement et Développement Durable (MEDD) de la RDC, Kinshasa, Democratic Republic of the Congo; 12 Forest Is Life, TERRA Teaching and Research Centre, Gembloux Agro Bio-Tech, University of Liège, Passage des Déportés 2, Gembloux, Belgium; 13 Q-ForestLab, Department of Environment, Faculty of Bioscience Engineering, Ghent University, Gent, Belgium; 14 Center for Ecosystem Science and Society, Northern Arizona University, Flagstaff, AZ 86011 USA; 15 School of Informatics, Computing and Cyber Systems, Northern Arizona University, Flagstaff, AZ 86011 USA; 16 INRAE, UMR ISPA, Villenave d’Ornon, France; 17 ISOFYS, Department of Green Chemistry and Technology, Ghent University, Gent, Belgium

**Keywords:** Forest ecology, Tropical ecology

## Abstract

We present a unique dataset of historical tropical tree phenology observations at two sites from different bioclimatic regions across the Congo Basin. We cover both the Atlantic Mayombe forest and the tropical forest in the central Congo Basin. To our knowledge this is the complete extant historical (1937–1957) phenology data across the Congo basin. The data contains ~10 million observations of 876 species, across 6339 individuals, and phenology metrics including leaf, flowering, and fruiting phenology. The data were recovered through expert transcription and validated community science based crowdsourcing. These data may provide a reference baseline and key information on how tree species are responding to a changing climate.

## Background

Tropical tree phenology affects the timing of critical ecosystem processes such as carbon, water and nutrient cycles^1^. As such, changes in tropical forest leaf phenology actively contribute to the modification of climate, from the local to global scale^2,3^. Tropical forests therefore play a significant role in the Earth’s carbon budget and strongly influence the regional and global climate^4–6^. In addition, the phenology of flowering and fruiting of trees connects to organisms across many trophic levels^7^ and is critical for the survival of pollinator and frugivore species. Frugivore species, in turn, are key in the propagation of tree species and have a profound impact on regeneration dynamics and potential of forests to recover after (anthropogenic) disturbances^7^.

For the central African ‘moist’ forest belt we have little process-based understanding of the forest’s phenological patterns. Previous studies have shown that species and even individuals seem to display a wide range of phenological behaviour and are potentially responding to different endogenous and exogenous cues^8,9^. A more complete characterization of the periodicity, synchrony and drivers of leaf phenology at the individual and species level is important to understand tropical tree phenology at the ecosystem scale, and to predict how stand-level phenology may alter with a changing climate^10^.

Given diverse species level responses it has been argued that using contemporary remote sensing solutions alone is insufficient for assessing tropical tree phenology^11^. This is specifically important for the Congo Basin, which stands out pantropically for being drier than the Amazon Basin or South-East Asia^12^, and exhibiting a bimodal rather than a unimodal seasonality^11,13^. Satellite analyses have suggested that the tropical evergreen state is not sustained year-round if mean annual precipitation is roughly below 2000 mm^12^.

At the same time, consistent and long-term historical observations of tropical tree phenology, across the Congo Basin or the tropics remain limited^10,14^. Despite these data limitations, it is increasingly important to understand how variations in climate have affected ecosystem functioning in the past to predict with more certainty how ecosystems will respond in the future. Few long-term high frequency phenology measurements are made of tropical tree species^14,15^ due to the challenges posed by such large species diversity^16^ and the sustained monitoring work involved over many years to decades needed to document patterns and processes within a rainforest ecosystem. Even less (baseline) data traces back to periods preceding large climate change induced disturbances experienced by these forests today^17^. It has been argued that the complexity of such forests necessitates ground-based long-term monitoring with a high coverage of species in tropical forests to understand both historical and current phenological behaviour and how phenology scales to a landscape level^10,15^.

Here, we present a unique dataset of historical tree phenology observations, using a consistent methodology, from the late 1930s to the late 1950s, across two sites in the Congo Basin. It covers both the Atlantic Mayombe forest and the tropical forest in the central Congo basin. To our knowledge, this is the complete extant historical phenology data across the Congo basin. The data contain ~10 million weekly to bi-weekly observations of 876 species, across 6339 individuals and various phenology metrics, including leaf senescence and turnover, flowering, and fruiting phenology. The data were recovered through expert transcription and validated community science based crowdsourcing and is augmented with stand level species distribution data where available. These data may provide key information on how sensitive seasonal dynamics in tree species and their co-dependent species, across trophic levels, are responding to a changing climate.

## Methods

### Study sites

**Fig. 1:**
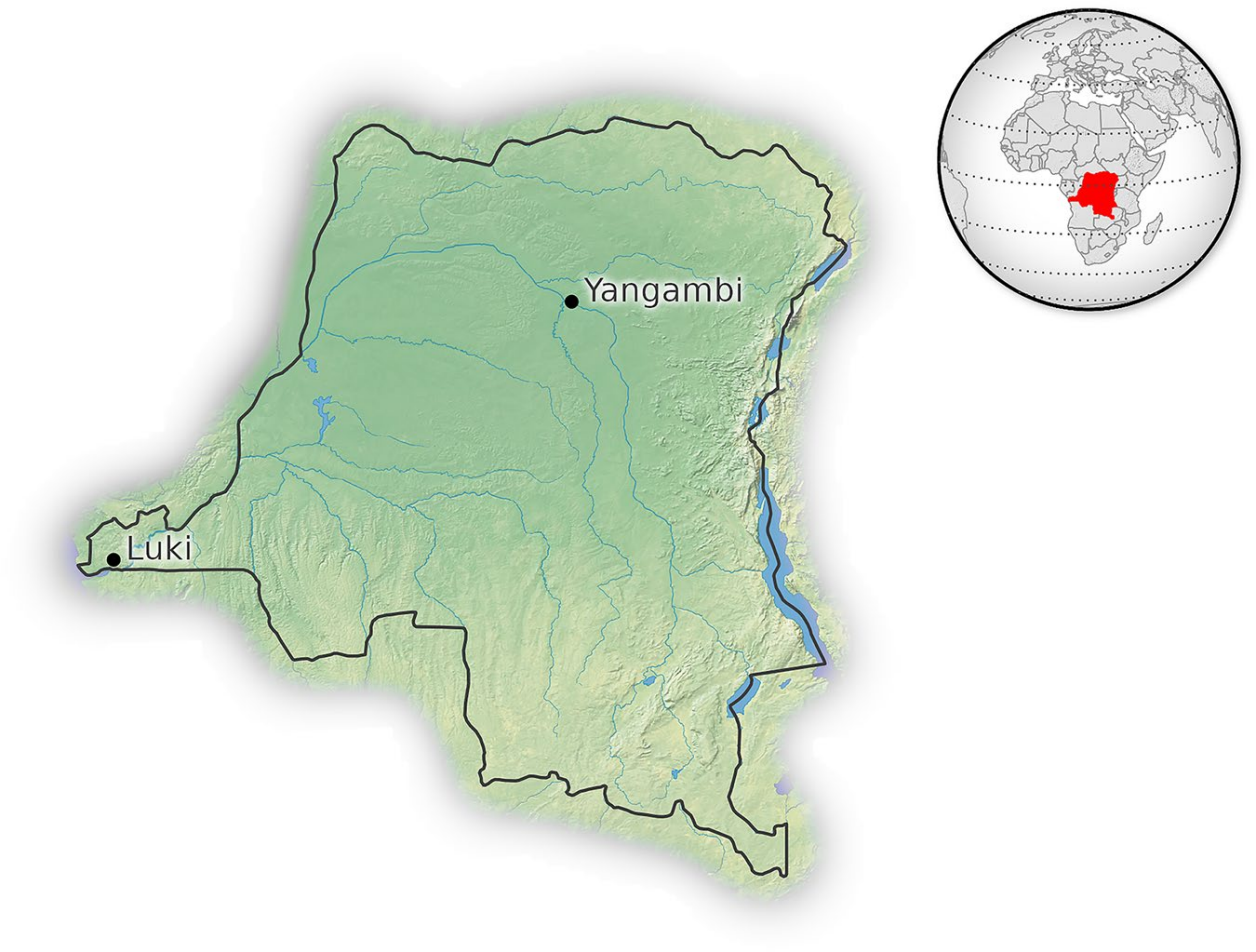
An overview topographic map of the Democratic Republic of the Congo (DRC) showing the low lying Congo Basin and indicating both research sites, Luki to the west and Yangambi to the east, respectively.

The Luki and Yangambi Research Stations are located in the Democratic Republic of Congo (DRC, see Fig. 1) and were historically part of an agriculture- and forestry-based research infrastructure project. The Luki and Yangambi research facilities were established in the 1920’s, and in 1933 integrated in the ‘Institut National pour l’Étude Agronomique du Congo’ (INEAC). The INEAC created and developed a network of 30–40 stations (depending on the time frame chosen), each with its own focus. Researchers in Yangambi for example focused on applied research and fundamental research in botany, climatology, tropical agronomy, forestry, and other disciplines. At the Yangambi and Luki research sites, moist semi-deciduous forest in both the central Congo Basin and the coastal Mayombe forests, respectively, continue to be studied. At both centres, the study of the forest flora, the establishment of scientific collections (exsiccata, seeds, seedlings, wood, pathogens and diseases), the observation of the rhythmic phenomena (phenology) such as leaf development, flowering and fruiting and the study of forest stands was central. The independence of the Congo in 1960 brought an abrupt end to the intensive research efforts and the accessibility of the accumulated expertise of the INEAC. Independence substantially impacted the operation and research of the institute in the following years and the institute was renamed to INERA in 1962. Today, INERA continues research at the agricultural centers across the DRC, where an impressive library with archived documents are stored in Yangambi with some duplicates in the State Archives of Belgium. At both sites, reserves were created in the 1940’s, and in the 1970’s they were declared protected Man and Biosphere reserves (MaB) by the United Nations Educational, Scientific and Cultural Organization (UNESCO).

The Yangambi MaB forest reserve is situated ~100 km downstream of the city of Kisangani in the central Congo Basin. Currently, the Yangambi forest reserve covers an area of 2,350 km^2^ just north of the Congo River with an average elevation of ~459 masl. The historical research facilities (including the herbarium, arboretum, climatological station) are located in the southwestern part of the reserve (N00°48′; E24°29′). The site currently also hosts the first eddy covariance tower in the Congo Basin (CongoFlux) and provides critical contemporary data on ecosystem fluxes^18^ and field based observations. Based on historical vegetation descriptions^19^, different types of vegetation are found in Yangambi. The most extensive part of the reserve consists of semi-deciduous old-growth forest. The floristic composition is highly diverse, although a high abundance of *Scorodophloeus zenkeri* Harms is generally found. Other species that are often abundant include *Cynometra hankei* Harms, *Dialium corbisieri* Staner, *Greenwayodendron suaveolens* (Engl. & Diels) Verdc., *Anonidium mannii* (Oliv.) Engl. & Diels, *Strombosiopsis tetrandra* Engl., *Prioria oxyphylla* (Harms) Breteler, *Celtis mildbraedii* Engl., *Prioria balsamifera* (Vermoesen) Breteler and *Pericopsis elata* (Harms) Meeuwen. Localized patches of the monodominant evergreen *Gilbertiodendron dewevrei* (De Wild) J.Léonard and of the evergreen dominant *Brachystegia laurentii* (De Wild) Louis J.Léonard are also present. In the valleys of the Congo River and tributaries, other vegetation types are found. The Yangambi reserve also contains sites with different levels of human intervention and secondary forests^20^. The central Congo Basin rainforests experience a weak seasonal climate with a bimodal periodicity of wet and dry seasons^21^ generated by the movement of the Intertropical Convergence Zone. Yangambi has the Af climate according to the Köppen classification with two slightly marked dry seasons, an average annual precipitation of 1837 mm, and mean annual temperature of 25.1 °C^22^. The reserve has two dry seasons with monthly precipitation lower than 150 mm, during December - February and June – July. Average temperatures are high and constant throughout the year with a minimum of ~24.2 °C in July and a maximum of ~25.5 °C in March. In this reserve, an increase in temperatures and temperature extremes has been observed since the 1960s. Since 2000, there has also been a notable change in rainfall patterns, with a shift towards a more seasonal climate characterised by longer dry periods and shorter, more intense rainy periods. This climate change is also having an impact on the composition and functioning of forest ecosystems and biodiversity in the Congo Basin^22^. The geology of the region consists of unconsolidated aeolian sedimentary sandy material of Pleistocene age, giving way to xanthic ferralsols^23,24^.

The Luki forest reserve is located 30 km north of Boma. The forest reserve covers ~ 330 km^2^ and spans a hilly landscape with altitudes varying from 150 to 500 masl over an extent of latitudes 5°30′-5°45′S, and longitudes 13°7′-13°45′E. The hilly terrain exposes the soil to heavy erosion and limits the formation of deep soils. Soils are ferralitic, acidic and of poor quality. Average annual temperature ~24.6 °C. Rainfall is seasonal with a pronounced dry season with only 60 mm of rain from June to September and an annual average of ~1180 mm. The hilly terrain in proximity of the ocean causes orographic cloud formation and keeps the relative air humidity high (>80%). A high air humidity prevents extreme water stress in the vegetation despite the pronounced dry season. For a detailed site description, including species composition, we refer to Couralet *et al*.^25^.

Phenological observations and forest inventories in the Congo Basin at Luki and Yangambi were made as part of wider forestry research efforts initiated by the INEAC to describe the phenology and stand dynamics of these two different forests from the late 1930s to the late 1950s^11,26^. Overall, some fifteen thousand (15 000) trees were regularly observed for years up to decades^27^ within the context of forestry research, of which less than half were observed for phenology research. We report here on phenological observations made on ~6000 monitored trees, including some lianas^28^, between the late 1930’s and late 1950’s across almost a thousand species, of which almost 500 trees can still be found growing to the present day.

### Data recovery

#### Observation protocols and records

**Fig. 2:**
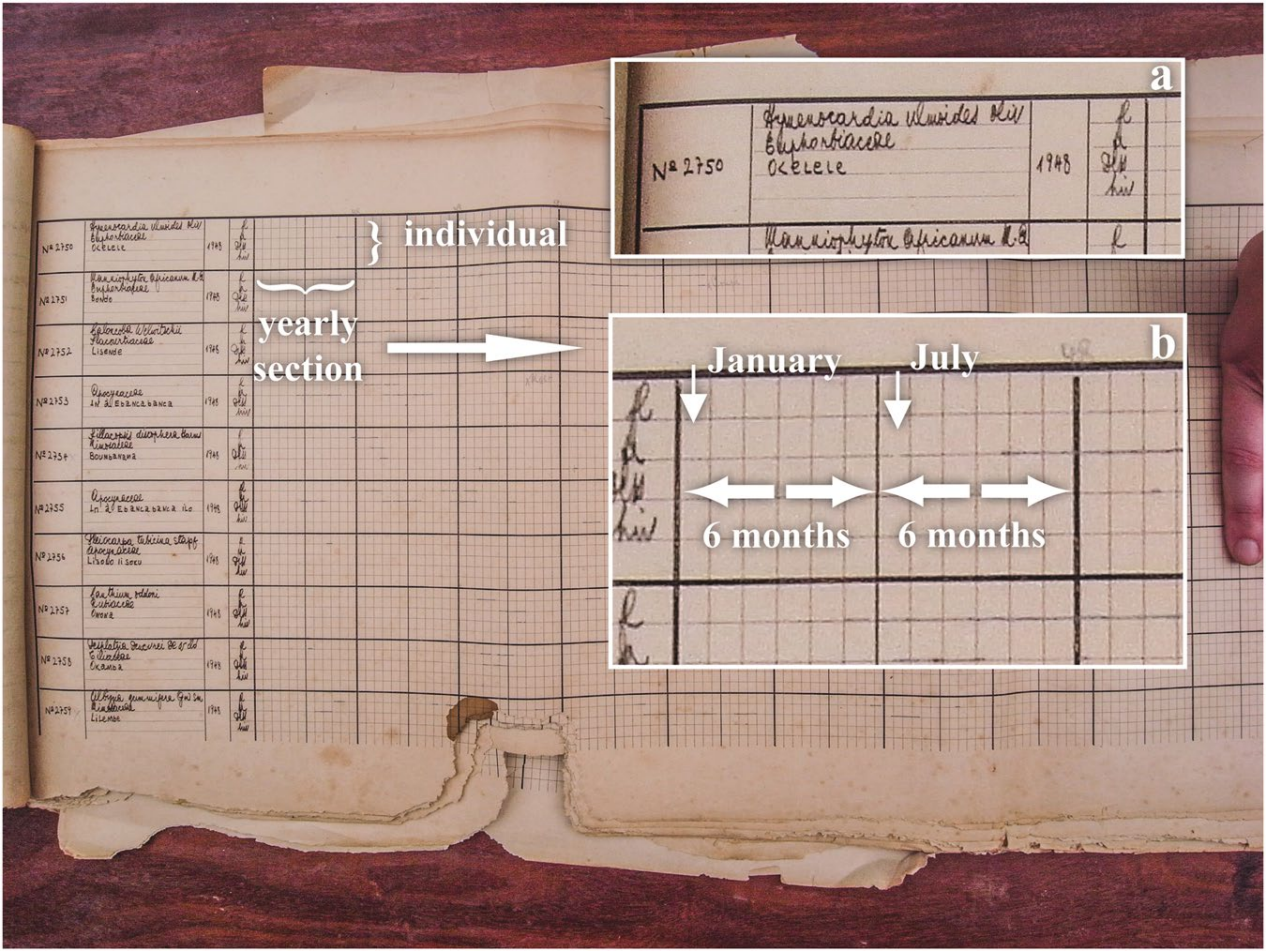
A summarizing data sheet of the historical phenological observations recovered at the Yangambi archives, at the time located at the herbarium library. Observations of 10 individual trees are shown (rows), identified with the tree’s ID number, species name, family name and local name (inset **a**). A year of observations is shown (inset **b**). Each consecutive year is marked by thick grid-lines. Vertical fine grid-lines represent one month, with half a year indicated with a vertical medium thick line. For each individual tree four phenophases are observed, indicated in French from top to bottom floraison (fl., flowering), fructification (fr., fruiting), dissémination (diss., seed dissemination) and hivernation (hiv., leaf senescence). Actual observations for each phenophase are indicated with pencil marks along the respective horizontal grid-lines. These pencil marks are drawn at a resolution of a quarter of a month, representing on average 7.2 days. The pencil markings of leaf senescence show a distinction of simple horizontal lines representing periods of leaf fall, while the tree remains bare, or cross-hatched lines during periods of leaf-fall and concomitant flushes of new leaves. The end of observation periods are often indicated with a cross, often combined with a reason, for example here the observation of the second individual stops during the second year due to mortality through tree fall (‘tombé par le vent’). Full summarizing data sheets cover ten individuals and ten years, with observations of individuals at times continuing on multiple sheets (a and b records). Note the data loss at the bottom due to rodents or insect damage on the summarizing table.

**Fig. 3:**
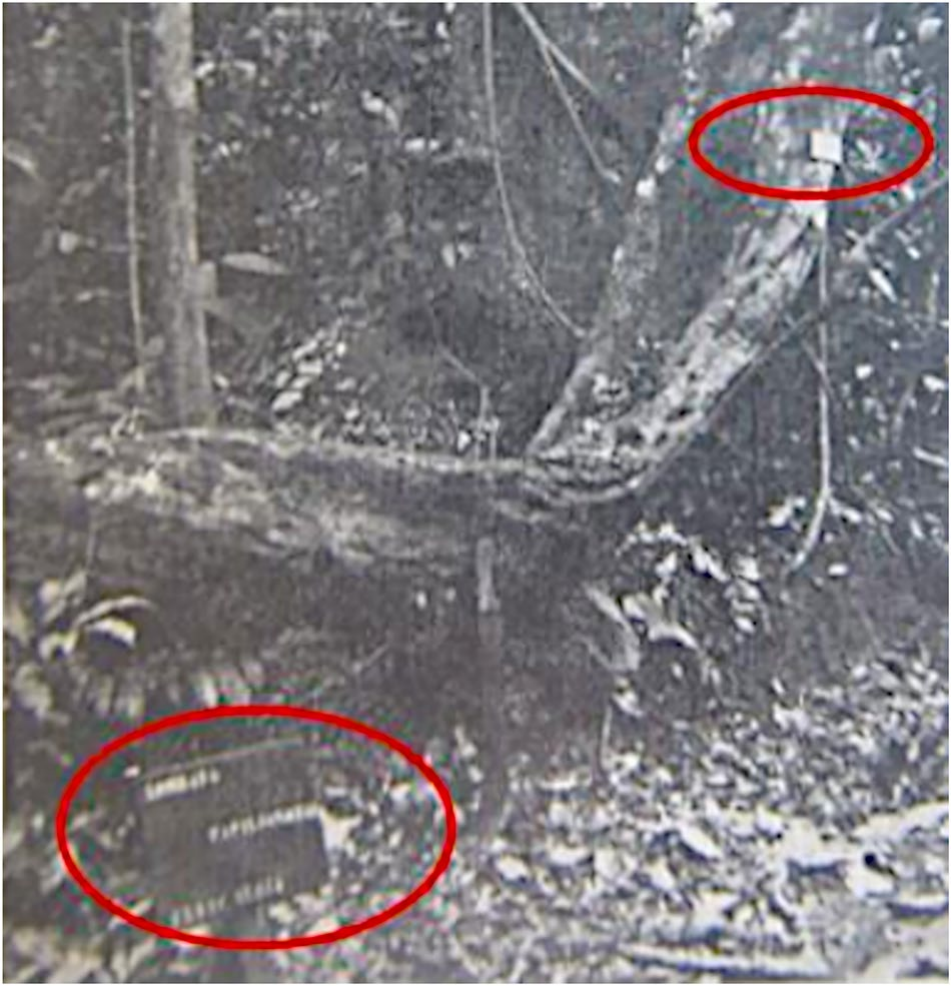
Photograph of the signs and ID plaques (in red ellipses) for trees along the Yangambi observation routes.

**Fig. 4:**
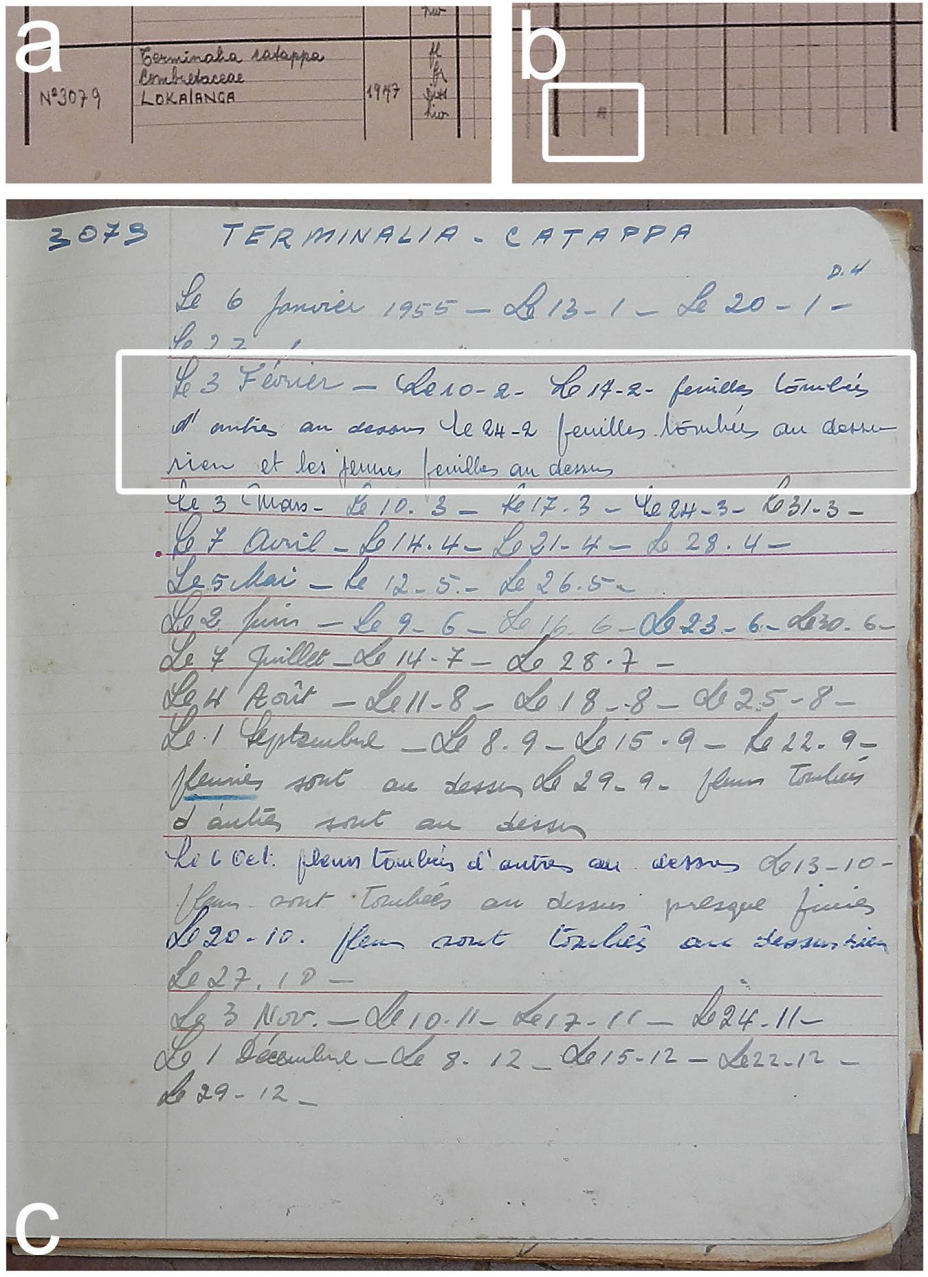
Cross referenced summary table of the row header (**a**) and content (**b**) with the original field notebooks containing the observations (**c**). In this sheet observations for February (Fevrier) describe “14 - 2 feuilles tombées d’autres au dessus”, or “14 - 2 fallen leaves with some on top”. The next week notes: “24 - 2 feuilles tombées au dessus rien et les jeunes feuilles au dessus”, which reads “24 - 2 fallen leaves, on top nothing and young leaves on top”. This confirms that crosshatched lines are leaf turnover events of semi-deciduous trees.

Historical data were recovered from a phenological study carried out between 1937 and 1956 in the Yangambi MaB reserve^28^. A total of 476 handwritten tables (Fig. 2) from the INERA (Institut National pour l’Etude et la Recherche Agronomique) herbarium at the Yangambi Research Station were digitized for archiving purposes. The sampling protocol we present here is based on the protocols found in the State Archives of Belgium^28^ or deduced from the raw data itself. Observations of tree phenology were made on a weekly schedule (approximately Monday to Saturday, with an interval of four weeks to a month), grouping trees by a fixed geographically constrained itinerary to limit travel time. Observational routes were followed, along which trees were flagged with bright blazes, signs (Fig. 3) or metal ID plaques (at Luki, see below), and observed by locally trained personnel familiar with the vegetation. At Yangambi mostly white paint on the trees, and a sign next to the tree was used. From the recovered historical descriptions we determined that every observer would observe roughly ~132 trees per day along their phenological route. Observations included the timing of flowering, fruit development and dispersion (fruit drop), leaf senescence (or turnover), and disturbances (tree death) which were written down in field notebooks and summarized in large tables (Fig. 4). Canopy leaf senescence was defined as a distinct period during which leaves fall and trees remain bare, while canopy turnover was defined as a period during which leaf-fall comes in peaks with concomitant flushes of new leaves (Fig. 4c). The observed trees are representative of the wide Yangambi region from the islands in the Congo river to the plateau to the north of Yangambi. The protocol at the Luki forest reserve followed the same observation schedule as applied in Yangambi. Here, 3750 woody plants were monitored from January 1948 until December 1957. The Luki dataset was manually transcribed by experts from the handwritten notebooks, similar to those used for the summary tables at Yangambi (see Fig. 4c). Due to data quality issues data was resampled to three observations per month. This data has been previously described and used by Couralet *et al*.^26^.

#### Transcription

The nature of the notation used on the summarizing data sheets (Fig. 2), using fine hand-drawn pencil lines overlapping fine table grid lines, made it impossible to process these images automatically. The Luki data was transcribed by experts, and previously described in literature^29^.

For the transcription of the Yangambi data we enlisted participants in the Jungle Rhythms community science project (https://www.zooniverse.org/projects/khufkens/jungle-rhythms). Participants were asked to annotate both the outline of the yearly table sections (if fully visible) and to annotate the observational features within the section. Each yearly section was presented to ten participants to improve accuracy of the annotations during post-processing.

**Fig. 5:**
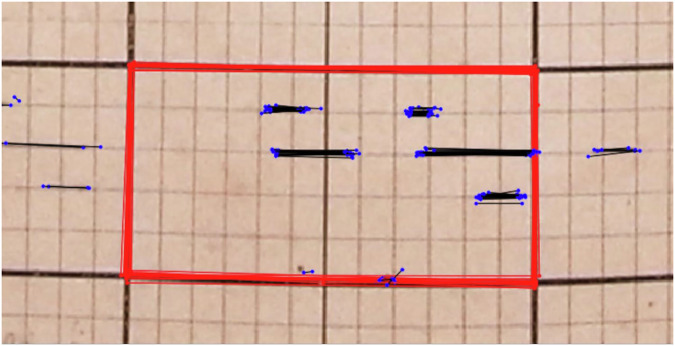
Example of a completed data annotation made through the community science project Jungle Rhythms. Volunteers were presented with sections of yearly observations outlined by the bold black lines. The edges delineating the year were marked using six coordinates represented by the red polygon (i.e. the start, middle and end at top and bottom of each section). Phenological observations were marked using line segments (black lines with blue dots). Each yearly section was presented to 10 independent participants. Median values for coordinates and line segments were used to determine the final annotation. Lines were reprojected using an idealized location, i.e. the underlying inferred grid.

The outline of each yearly section (six coordinates; i.e. the start, middle and end at top and bottom of each section) provided the general orientation of the section within the image, allowing the annotations to be translated into true data compensating for skewness and warping in the image using simple geometry. The weeks in which certain phenological events occur are calculated, using basic geometry of projecting lines onto an idealized grid, and the entire record for each individual tree is compiled. Median values (majority vote across ten community science annotations) were used to delineate each phenology feature, i.e. the extent of continuous phenological events. The Yangambi data record was not free of damage due to paper decomposition, in particular on creased edges, as well as rodent or insect infestation. Years which could not be clearly outlined (with six intersection points, Figs. 2, 5) were not transcribed, and are omitted from the data record (see usage notes).

### Ancillary data, species identification and data harmonization

To support further analysis we transcribed supporting stand level species distribution data at Yangambi from Pierlot (1966, pages 264–299, plots 20–22). This includes sampling plot data with respect to species composition and basal area as diameter at breast height (DBH, as circumference at 1.5 m) in incremental steps of 20 cm from >30 cm onward across three forest transects. Tree circumferences were converted to basal area per hectare (ha) per species.

Species names were consolidated across all datasets and were verified by the authors using the African Plant database (http://africanplantdatabase.ch/), through world flora online (https://www.worldfloraonline.org/). For the phenological data, additional cross references with the Meise Botanic Garden collection (https://www.botanicalcollections.be/) were made, since most of the ‘observed trees’ were sampled and herbarium vouchers are available in the Meise herbarium, thus a retroactive quality check on identification was possible. If uncertainty existed on the species identification of an individual (for example by unclear hand-writing or data loss through time), this individual was marked in the dataset quality assurance (qa) field (‘species_qa’, see below). All observation data was harmonized to a 365 day-of-year (DOY) scale by mapping a year with 36 or 48 observations, for Luki and Yangambi respectively, to a consistent 365 days (e.g. an observation at week one in Yangambi would correspond to DOY 7).

## Data Records

**Fig. 6:**
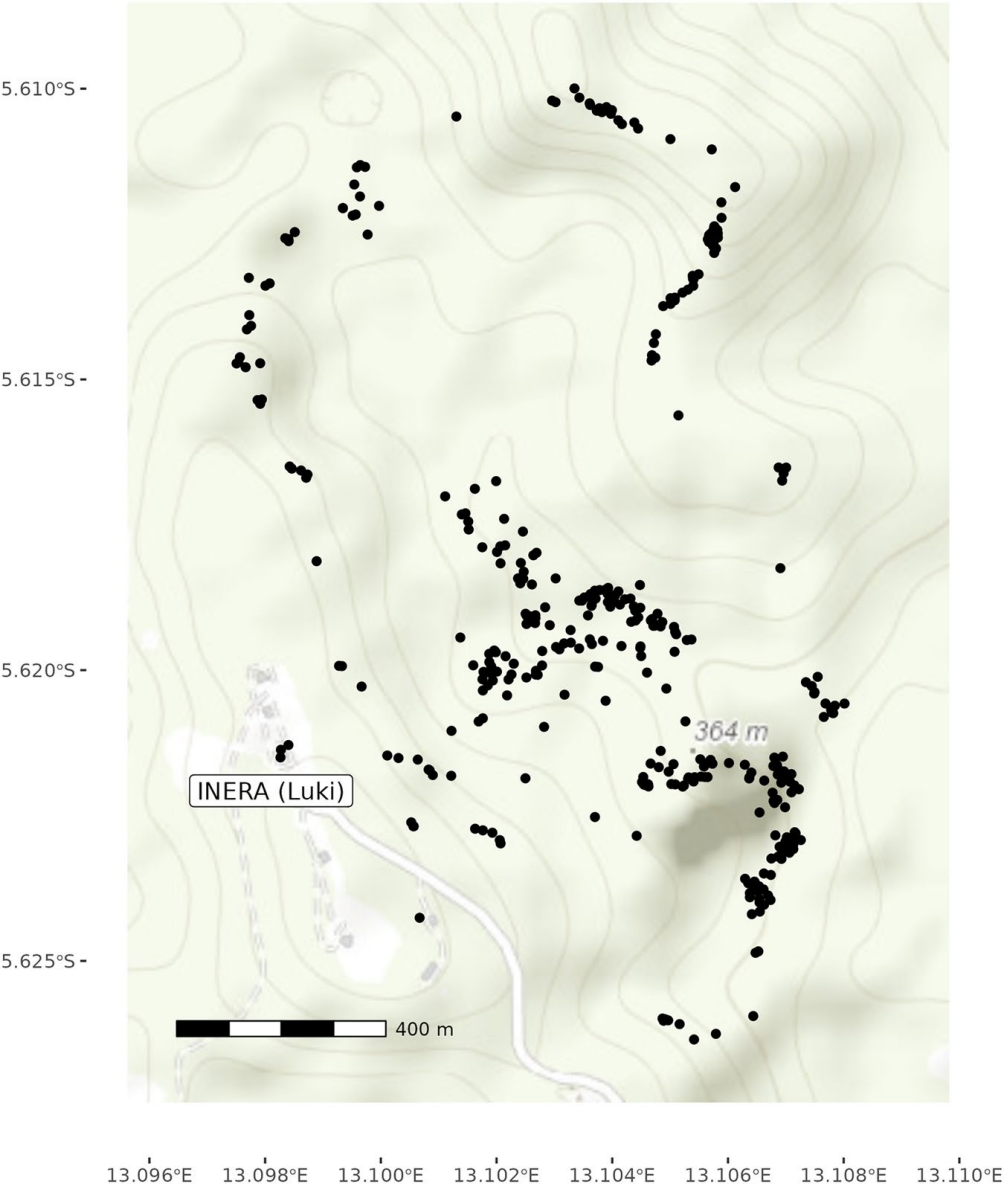
Overview map of at the Luki site showing the locations of individual trees (as a black dot). [map data from OpenStreetMap under the Open Database License].

The complete recovered phenological dataset for both Luki and Yangambi covers observations between 1937 and 1957 of 6339 individual trees, and is available on Zenodo^30^ (10.5281/zenodo.16789580). The Yangambi site represents 2396 individual trees and 15269 site years, ~3.8 million individual observations, covering 826 species. The Luki site represents 3943 individual trees, 42586 site-years and ~6.8 million individual observations covering fewer (155) species with higher replicate individuals. Where possible, exact coordinates of trees are provided, however this does not guarantee that the tree is currently alive. When no exact coordinates are known, e.g. all of the Yangambi data, we used the coordinates of the Luki and Yangambi MaB reserve headquarters. A separate data field ‘coord_type’ is used to mark site and individual (tree) based coordinates (see Table 1, Fig. 6).

**Table 1:** Overview of the CSV/RDS data fields, data types and an example record and the categorical values with their possible options.

table column name	data type	example	categorical values
site	character	Yangambi	Yangambi, Luki
latitude	numeric	0.76236	
longitude	numeric	24.4629	
coord_type	character	site	site, individual
elevation	numeric	442	
id	character	3095	
year	numeric	1948	
week	numeric	1	
doy	numeric	8	
historical_common_name	character		
historical_genus_species	character	Erythrina abyssinica	
family	character	Leguminosae	
genus	character	Erythrina	
species	character	abyssinica	
species_qa	character	accepted	accepted, unknown, unresolved
phenophase	character	leaf_turnover	leaf_turnover, leaf_dormancy, flowers, fruit, fruit_drop
value	numeric	0	0, 1

The phenology data is provided as a single serialized and compressed (.tar.gz) CSV flat file, and a binary compressed ‘R’ serialized RDS file, describing all phenological phases and their timing using the format described in Table 1. The data is provided as a long format with a row representing a single observation. Fully accepted species names are marked as “accepted” in the ‘species_qa’ field of the datasets, unknown species (due to inconsistent species names) are marked as “unknown”, species which have issues with respect to their naming are marked “unresolved”. Data users can filter based upon the status of species names.

In addition to the phenology data itself, life-history notes are included in a separate file (Table 2). These life history notes include observations made at the end, or occasionally during, a full phenology record of a tree. These data did not correspond to the binary, presence absence, data of the five indicated phenophases. These notes were either transcribed in full for the Luki site, or extracted from hashtag data in the community science forums for Yangambi. We note that for Yangambi the hashtag data was not part of a formal workflow and lacks quality control (multiple views per yearly section). The stand level inventory data by Pierlot^31^ includes data on the species, circumference (from 30 cm onward, in 20 cm increments), and overall species count per plot (Table 3). The data was transcribed and is provided as a CSV flat file.

**Table 2:** Overview of the data fields, data types and an example of values in the life-history notes of both the Luki and Yangambi data.

table column name	data type	example
site	character	Yangambi
id	numeric	3137
family	character	Menispermaceae
genus	character	Jateorhiza
species	character	macrantha
notes	character	coupe

**Table 3:** Overview of the CSV data fields, data types and an example value of the stand level species distribution and basal area data included in Pierlot 1966.

table column name	data type	example
inventory	character	Inventory 20
nr	numeric	2
circ_class	numeric	3
count	numeric	3
species_qa	character	accepted
genus_species	character	Combretum lokele
latitude	numeric	0.833333
longitude	numeric	24.5
elevation	numeric	450
area_ha	numeric	40

## Technical Validation

Quality control and validation of the data produced through citizen science was performed using an independent validation dataset made by the authors for ~2% of the data. Data was chosen to overlap with ongoing research questions and was therefore unbalanced (non-random) across species or phenophase occurrences. All phenophases were directly transcribed into data indicating the exact weeks of the observation. We used the F-score^32^, or the harmonic mean of precision and recall, to assess the accuracy between the citizen science recovered data and expert transcription (Table 4). An F1 score of 1.0 indicates a perfect precision and recall. Across all phenophases we find an F1 score of 0.8 ± 0.19 and accuracy of 0.98 ± 0.001. However, the average values are negatively influenced by smaller sample sizes of leaf dormancy and turnover in the unbalanced validation dataset. As the protocol for transcription was the same for all phenophases in a yearly section (Fig. 2a) it seems unlikely that differences between phenophases are due to consistent errors in the community science transcriptions.

**Table 4:** Classification validation statistics in the form of the F1 score and accuracy metrics.

Phenophase	F1	Accuracy	Positive observations (out of ~55 K)
flowering	0.936	0.984*	6736
fruiting	0.956	0.976*	15376
fruit_drop	0.887	0.989*	2612
leaf_dormancy	0.513	0.993	467
leaf_turnover	0.72	0.992*	590

Values where the accuracy is higher than the F1 score which would be acquired by classifying any observation a fixed value are indicated with*.

At Yangambi, given the lack of the full protocol, it is hard to know if the data presented is the full scope of all work ever conducted. We know that the emphasis on species changed over time when observations were transferred from the botanical division (Division Botanique) to the forestry department (Division Foresterie), which retained mostly commercial species toward the end of the observation period. Many trees with observations starting in 1937 were cut for wood biological studies and received a wood biology database (Tw) number of the Royal Museum for Central Africa (https://www.africamuseum.be/). These changes over time through anthropogenic or natural causes are noted by both the non-sequential nature of trees in the weekly planning (Fig. 2) and the recovered life history notes. However, we do acknowledge that, as far as we know, we transcribed all available archived summary tables. Assessing the full coverage of the data would require the re-evaluation of all long-form handwritten notebooks (Fig. 4).

Unlike studies using a similar protocol at the Luki MaB reserve, no remaining metal tags or exact phenology observation routes could be found in Yangambi. Yet, descriptions of the locations suggest trees covered the wide Yangambi region (see literature by Capon^28^, and additional meta-data). From literature, i.e. Capon, we identified nine key areas, mainly (1) Parc Isalowe (‘Parc Forestier’), (2) along the river Isalowe, (3) around the building of the forestry division, (4) at the phyto-technical department (current abandoned buildings of the IFA), (5) near experimental sites, (6) along the river Lusambilla, (7) along roads to Yangambi from Kisangani, Isangi or NGasi (‘Arbres éloignées’ or far removed trees) (8) on islands of the Congo river, and (9) in front of the Yangambi reserve (Tofende, Lomondje, Tutuku, Esali). This wide distribution has a considerable importance with respect to phenology as it mediates both soil properties and water availability during the dry season. In an early study based on the Yangambi data by Capon^28^ these geographic differences were already noted. For example, *Lannea welwitschii* fruits appeared continuously on the islands in the Congo river, while showing a far shorter cycle in other locations. Observations of fruiting were limited to those species which drop fruit. Capon^28^ acknowledges that observations near water or fruits desirable by humans or animals (in large quantities) might be biased as well. Similarly, they further noticed that spotting flowers on larger trees took considerable practice by observers as illustrated by historical communications to INEAC management complaining about the tedious nature of these observations. Given the wide range of locations and differences in soil and soil water conditions this might influence analysis. Where exact coordinates are missing the reported coordinates in the dataset are those of the current INERA headquarters. Users should be mindful of these limitations.

Data missingness, as in the absence of certain months or years, should be interpreted as physically unavailable data if the time series continues throughout. Missing observations can be caused by the physical destruction of the tables where the records were folded and cracked, damage from rodents, to year-on-year changes in observation schedules due to world historical or local events (e.g. World War II) or shifts in institutional interests. Years with all values set to zero (0) did not record any phenological events. Poorly annotated species data in the tables leads to unknown species information. We encourage users to use the “species_qa” flag to filter out “unknown” or “unresolved” species from the dataset as they see fit. Given these limitations we encourage users to use Yangambi phenology statistics in seasonal or yearly aggregate when possible, or cross validate these values with the provided images and or additional (local) species information to remediate these usage issues. In absence of a clear marking of the tree’s death it is hard to establish where phenological records end. We recommend considering the extent of any positive phenophase values to indicate continuous observations throughout or the last year with any phenophase values as the final year of observation. We further note that Yangambi data ran on a four week to a month schedule, while Luki was gathered on a 10-day (3 intervals) per month. Harmonization to a DOY scale allows for a direct intercomparison within the context of modelling efforts.

## Data Availability

The manuscript’s database is made available as a Zenodo Digital Repository (10.5281/zenodo.16789580). The original digitized tables, and the citizen science (raw) transcriptions are available as zipped archives (10.5281/zenodo.14906306 and, 10.5281/zenodo.14900505 respectively).

## Code availability

The code and workflow used during ‘Jungle Rhythms’ community science project are available as an R project (10.5281/zenodo.7650294).
